# Mere Expectation to Move Causes Attenuation of Sensory Signals

**DOI:** 10.1371/journal.pone.0002866

**Published:** 2008-08-06

**Authors:** Martin Voss, James N. Ingram, Daniel M. Wolpert, Patrick Haggard

**Affiliations:** 1 Sobell Department of Motor Neuroscience, Institute of Neurology, London, United Kingdom; 2 Department of Psychiatry, St. Hedwig Hospital & Charité University Hospital, Humboldt University, Berlin, Germany; 3 Department of Engineering, University of Cambridge, Cambridge, United Kingdom; 4 Institute of Cognitive Neuroscience and Department of Psychology, London, United Kingdom; Victoria University of Wellington, New Zealand

## Abstract

When a part of the body moves, the sensation evoked by a probe stimulus to that body part is attenuated. Two mechanisms have been proposed to explain this robust and general effect. First, feedforward motor signals may modulate activity evoked by incoming sensory signals. Second, reafferent sensation from body movements may mask the stimulus. Here we delivered probe stimuli to the right index finger just *before* a cue which instructed subjects to make left or right index finger movements. When left and right cues were equiprobable, we found attenuation for stimuli to the right index finger just before this finger was cued (and subsequently moved). However, there was no attenuation in the right finger just before the left finger was cued. This result suggests that the movement made *in response* to the cue caused ‘postdictive’ attenuation of a sensation occurring *prior to* the cue. In a second experiment, the right cue was more frequent than the left. We now found attenuation in the right index finger even when the left finger was cued and moved. This attenuation linked to a movement that was likely but did not in fact occur, suggests a new expectation-based mechanism, distinct from both feedforward motor signals and postdiction. Our results suggest a new mechanism in motor-sensory interactions in which the motor system tunes the sensory inputs based on expectations about future possible actions that may not, in fact, be implemented.

## Introduction

When a part of the body moves, the sensations evoked by a probe stimulus to that body part are attenuated [1], [2]. Sensory-motor attenuation is a robust and widespread phenomenon in motor control, occurring for somatosensory, visual and auditory systems [3], [4]. It may serve to prevent overload due to the large amount of afferent information generated during action, or to highlight novel external events unrelated to one's own action [5].

Two quite different mechanisms have been proposed to explain this effect, based on efferent and afferent processes respectively. According to the efferent explanation, the motor system may modulate activity evoked by incoming sensory signals. Several studies have considered at what level in the motor hierarchy the efferent signals responsible for sensory attenuation may occur. In one computational model, efference copy of voluntary motor commands cancels afferent feedback resulting from the action. However, when the motor command output from the primary motor cortex was artificially delayed, sensory attenuation nevertheless occurred at the time of the intended rather than the delayed movement [6]. Therefore, sensory attenuation must arise from stages in the motor processing hierarchy upstream from the dispatch of motor commands from the cortex [7]. For example, attenuation was also found for a short period immediately after a simple movement was cancelled by a NoGo signal [8]. We therefore suggest that merely preparing, but not executing, a motor command, may be sufficient for sensory attenuation. Attenuation due to preparation might also explain why somatosensory evoked potential amplitudes are reduced prior to voluntary action [9]–[11].

According to an alternative afferent explanation, reafferent sensations from body movements may mask the sensory probe. In support of this view, passive movements were reported to produce a similar amount of sensory attenuation to active movements. This was true even when probe stimuli were applied before movement onset [12], suggesting a backward masking effect. SEP studies confirmed that cortical activity is not modulated prior to passive movements [13], again suggesting that the mechanism is postdictive. In general, efferent and afferent mechanisms of sensorimotor attenuation cannot easily be distinguished on the basis of the time at which each occurs.

The experiments in this study were designed to identify whether sensory attenuation prior to movement is afferent or efferent in origin, and at what level of the motor hierarchy it may occur. Rather than relying only on timing to distinguish afferent from efferent mechanisms, we manipulated subjects' expectancy about which movement they would make in a cueing task. This allowed us to dissociate preparation of efferent motor commands, from their execution, and from reafferent information about the actual movement.

## Results

**Figure 1:**
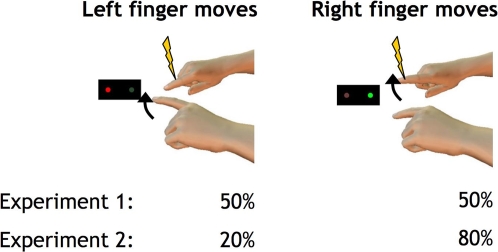
Experimental setup for both experiments: Subjects are moving either the left or right index finger in response to a visual go signal (red cue – left finger, green cue – right finger; assignment of cues randomised over subjects). At various intervals relative to the visual cue (−200, −100, −50 and triggered by movement onset in experiment 1, at −50 ms in experiment 2), the right finger was stimulated with a brief electrical shock of varying intensities (yellow arrow), the left little finger received a simultaneous shock (not shown) of a fixed intensity (150% detection threshold for each individual subject) which served as the reference in the forced-choice paradigm. In experiment 1, visual cues appeared equiprobable for both left and right finger movements in random order; in experiment 2, right cues were 4 times more probable than left cues.

Subjects compared the intensity of a brief electrical stimulus applied to the right index finger with a simultaneous reference stimulus on the left little finger (for a schematic illustration of the experimental setup see Figure 1). The strength of the test stimulus was adjusted (see experimental procedures) to find the point of subjective equality (PSE). PSEs measured at rest before and after the experiment, were averaged to give a baseline. PSEs in experimental conditions were expressed as percentage changes from this baseline, and used to measure sensory attenuation.

### Experiment 1: Sensory attenuation

**Figure 2:**
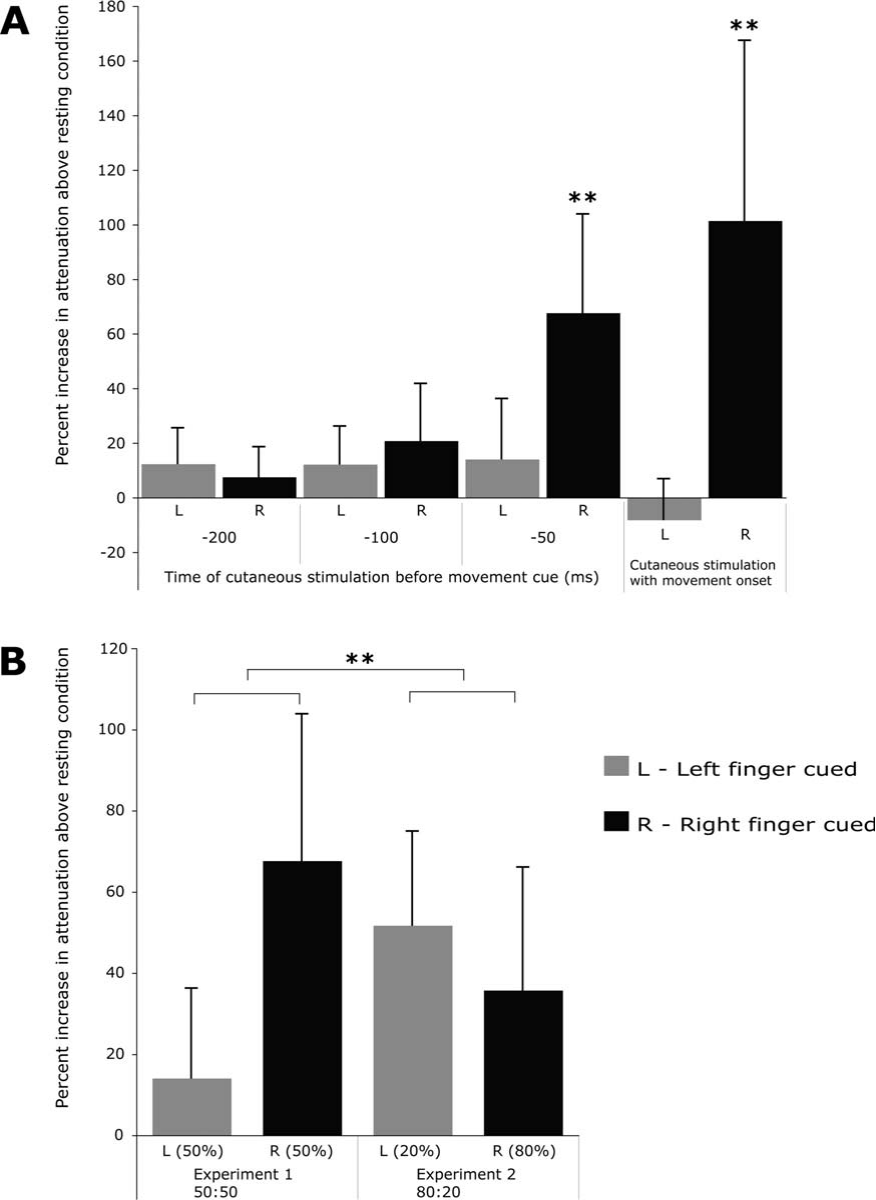
Percent increase above resting condition in the “point of subjective equality” (PSE) for a probe stimulus applied to the right index finger as compared to a reference stimulus to the left little finger. A) shows results from experiment 1 for left (grey bars) and right finger movements (black bars), when stimuli were delivered 200, 100 and 50 ms prior to a cue instructing which finger to move or together with movement onset with equiprobable chance of left and right cues. Asterisks indicate highly significant (p<0.001) changes as compared to the baseline (resting) condition (two-tailed t-tests), which only occurred when the right finger was subsequently cued. B) shows results from experiment 1 (left side) and experiment 2 (right side) in which right cues occurred 4 times more frequently than left cues (“80:20”). A highly significant interaction (ANOVA interaction F1,14 = 17.22, ** = p<0.001) arose because attenuation in the right finger occurred also on rare left-cued trials in the 80:20 group only, suggesting a new expectation-based mechanism of sensorimotor attenuation.

In experiment 1, subjects heard a series of 3 tones ending with a visual go signal equiprobably (“50:50”) instructing either a left or right index finger extension. PSEs were measured for electrical stimuli occurring at 200, 100, 50 ms before the cue (approximately 500, 400 and 350 ms respectively before movement onset) and at movement onset. We found a significant and strong attenuation of stimuli to the right finger both at movement onset (increase in PSE above baseline by 101.4%, p<0.001, two-tailed t-test) and also 50 ms *prior* to a right cue (67.6 % increase PSE p<0.001). Attenuation 100 and 200 ms before the cue did not reach significance. Cueing and moving the left finger never attenuated sensation on the right finger (Figure 2 A).

### Experiment 2:

In experiment 2, a new group of subjects responded as before, but right cues occurred 4 times more frequently than left cues (“80:20”). This encouraged subjects to prepare a right finger movement in advance, and indeed, reaction times were faster for right finger movements than left finger movements, in experiment 2, but not experiment 1 (ANOVA interaction F1,14 = 17.22, p<.001, follow-up t-test left vs right in the −50 ms condition: p = 0.0079 for experiment 2, p = 0.44 for experiment 1).

Probe stimuli to the right index finger were now always given 50 ms before the cue. Again, probes before right cues were significantly attenuated (PSE increase by 35.7%, p = 0.015), confirming postdictive attenuation. Interestingly, and in contrast to experiment 1, we now also found significant attenuation of probes to the right finger even on the 20% of trials where the subsequent cue instructed a left finger movement (PSE increase by 51.7%, p = 0.01). We compared the PSEs 50 ms prior to a cue using ANOVA with factors of experiment (50:50 vs. 80:20) and finger cued (left vs. right). This showed a significant interaction between cue probability and finger cued (F1,14 = 12.11; p = 0.004), but no significant main effects. Figure 2B suggests that this interaction arose because attenuation in the right finger occurred on rare left-cued trials in the 80:20 group but not the 50:50 group. Follow-up simple effects testing confirmed this impression. This showed that the increased expectation of a right cue in the 80:20 group caused attenuation even when the left finger was cued (p = .008). Conversely, attenuation when the right finger was cued did not differ significantly between the two experiments.

Because sensory suppression depends on movement parameters such as movement amplitude and velocity [1], we assessed whether the differences between conditions in sensory suppression might merely reflect accidental differences in the parameters of the right index finger movement. In experiment 1, the averages (and standard deviations across subjects) of movement amplitude, maximum speed and duration for the right finger were 3.9±1.4 cm, 34±8.6 cm/sec and 0.35±0.019 sec in the movement-triggered condition, and 4.04±1.2 cm, 37.4±9.6 cm/sec and 0.15±0.06 sec in the condition where stimulation was delivered 50 ms prior to movement onset. In experiment 2 the averages (and sd across subjects) of movement amplitude, maximum speed and duration were 5.76±1.7 cm, 54.6±15.7 cm/sec and 0.16±0.05 sec.

To assess whether the variations could have some effect on PSEs, we combined the data from both experiments, and used analysis of covariance (ANCOVA) to investigate whether any underlying relationship existed between PSE values and movement amplitude, velocity or duration. None of these parameters showed a significant relation with PSE (all p>0.38), and none explained more than 2% of the variance in PSE values across subjects and conditions.

## Discussion

Our results showed evidence for postdictive attenuation (experiment 1), but also evidence for predictive attenuation when subjects could prepare the movement in advance, based on their expectations about the cue (experiment 2).

In experiment 1, attenuation for a probe stimulus occurring prior to the cue instructing the subject *which* finger to move, can be explained neither by feedforward motor command signals, nor by higher-level motor preparation. Explanations based on the motor command can be ruled out because the probe was attenuated even before the cue was given. Explanations based on preparation can be ruled out because left and right cues occurred randomly and equiprobably, so subjects had no basis for preparing one movement rather than the other. Therefore, we conclude that the actual movement executed retrospectively affects perception of probes preceding the cue, presumably by a form of backward masking. In our study, the average reaction time for the right finger was 325 ms, and we observed postdictive attenuation for probes occurring 50 ms, but not 100 ms, prior to the cue. Therefore, we conclude that postdictive attenuation can extend backwards in time for up to 375 ms. This is at least twice the window previously reported for such postdictive effects. For example, previous somatosensory studies reported attenuation for a period of up to 150 ms before both active and passive digit movement [12], [14]. We suggest that postdictive mechanisms play a more important role in sensorimotor attenuation than previously thought.

In experiment 2, we observed attenuation of a probe to the right finger, even when the left finger was cued, and subsequently moved, while the right finger did not move. This cannot be attributed to retrospective afferent masking or to motor commands, since the right finger did not move. Instead, we suggest that subjects in experiment 2 expected a right cue and accordingly prepared to make a movement of the right finger. This was confirmed by their shortened reaction times. We show that this preparation was itself sufficient to cause sensory attenuation. Thus, the mere *preparation* of action has a structuring effect on somatosensory perception, even when the motor command is not actually executed. Preparation clearly involves several component processes, including anticipatory selection of appropriate motor responses [15], and also appropriate shifts of attention [16]. The direction of sensory modulations in our study shows that the results reflect motor preparation, and not preparatory shifts of attention: attention typically enhances, rather than attenuates perception [17], [18].

Our result provides the first evidence for predictive sensory attenuation based on higher-level motor preparation alone, excluding explanations based on both motor command and (re-)afferent mechanisms. A previous study [8] compared sensory attenuation immediately *after* go or nogo cues. There, however, the focus was not on preparation prior to these cues, but on the differences between development of sensory attenuation following go cues, and the release of attenuation after nogo cues. Attenuation *prior* to go/nogo cues was not tested, nor was there any independent evidence that subjects prepared actions in expectation of go cues. Therefore, that study did not provide clear evidence that motor preparation is sufficient for attenuation. Sensory attenuation is normally explained in terms of sensory predictions based on the motor command being compared to actual sensory feedback. Here, we suggest that such predictions occur also at higher, preparatory levels in the motor hierarchy. Attenuation is therefore a general principle of motor sensory interaction, not just a specific physiological mechanism.

## Materials and Methods

### Subjects

24 right-handed subjects (10 male, 14 female, age range 21–28 years) gave written informed consent to participate in the study and were naïve to its purpose. The study was conducted in accordance with the Declaration of Helsinki and was approved by the National Hospital for Neurology and Neurosurgery and the Institute of Neurology Joint Research Ethics Committee.

### Cutaneous stimulation

Brief electrical cutaneous stimuli were generated by an electrical nerve stimulator (Stanmore stimulator, research device designed and developed by the medical physics department, UCL, London, UK) and applied to left little and right index finger simultaneously using stainless steel ring electrodes (SLE Ltd., Surrey, UK). The stimulus intensity was varied by modulating the pulse width between 0.02 and 0.4 ms while the current intensity was kept constant at 10 mA.

### Experimental procedure

Subjects were seated in a comfortable chair with both their arms resting on a table in front of them. Prior to the experiment, the point of subjective equality (PSE) for simultaneous stimuli applied to the left little (reference) and the right index finger was determined at rest: Stimulus intensity to the left finger was set to a fixed pulse width (150 % of its detection threshold determined prior to the procedure), while the pulse width of stimuli applied to the right finger was varied across trials. A two alternative forced-choice paradigm was used in which subjects had to report which of the two stimuli felt stronger. The next stimulus intensity for each condition was chosen from a uniform random distribution bounded by the 1 % and 99 % points on the fitted psychometric logistic curve. 50 valid trials were collected for each condition to determine the PSE for the perception of the electrical pulses. The same procedure was repeated at the end of the experiment and the average was used as the baseline condition.

Throughout the experiment, subjects fixated a black box with two LEDs, one green and one red, 5 cm apart from each other on a horizontal line. On each trial, three consecutive tones were played with an inter-tone interval of 800ms. On the third tone, one of the two LEDs instructed the subject to make either a left or right index finger lift. Movements had to be initiated within a 400 ms time-window after the third tone. Movement onset was monitored using an Optotrak 3020 optical infrared tracking system (Northern Digital, Waterloo). Subjects were informed if their movement occurred too early (i.e. before the cue indicating which finger to move was presented) or too late (i.e. more than 400 ms after the cue) and such trials were discarded from analysis.

In experiment 1, 8 subjects received brief cutaneous stimuli either 50 ms before the movement cue or with movement onset. The PSEs for both stimulus timings were determined in parallel by randomly selecting each trial from two independent forced-choice search procedures. A further 8 subjects received stimuli 100 or 200 ms prior to the cue. Left and right cues were random and equiprobable.

In experiment 2, 8 subjects experienced cutaneous stimuli 50 ms prior to the movement cue. Movement cues were randomised, but right cues now occurred 4 times more often than left.

### Data analysis

For each condition, the subjects' individual trial responses (left or right stimulus perceived stronger) were fitted with a logistic function according to a maximum-likelihood procedure. This function was then used to estimate the Point of Subjective Equality (PSE), defined as the intensity of a stimulus to the right index finger which would feel as strong as the reference stimulus to the left little finger. PSE values in each condition were first normalised to the rest condition, and the effect of each condition was expressed as a percentage *increase above* the rest level. Statistical comparisons were made between conditions using two-tailed paired t-tests; and factorial ANOVA. The reaction time on each trial was measured as the interval between visual cue onset and the first time when index finger movement recorded by Optotrak exceeded a velocity of 5 cm/s.
